# Functional connectivity between tumor region and resting-state networks as imaging biomarker for overall survival in recurrent gliomas diagnosed by *O*-(2-[^18^F]fluoroethyl)-l-tyrosine PET

**DOI:** 10.1093/noajnl/vdaf023

**Published:** 2025-01-29

**Authors:** Michel Friedrich, Jan-Michael Werner, Joachim P Steinbach, Michael Sabel, Ulrich Herrlinger, Marc Piroth, Gabriele Stoffels, Christian P Filss, Philipp Lohmann, Nadim J Shah, Maximilian I Ruge, Felix M Mottaghy, Roland Goldbrunner, Karl-Josef Langen, Gereon R Fink, Martin Kocher, Norbert Galldiks

**Affiliations:** Institute of Neuroscience and Medicine (INM-3, INM-4, INM-11), Forschungszentrum Juelich, Juelich, Germany; Department of Neurology, Faculty of Medicine and University Hospital Cologne, University of Cologne, Cologne, Germany; Department of Neuro-Oncology, University Hospital Frankfurt - Goethe-University, Frankfurt, Germany; Department of Neurosurgery, Heinrich Heine University Medical Faculty and University Hospital Düsseldorf, Düsseldorf, Germany; Center of Integrated Oncology Aachen Bonn Cologne Duesseldorf (CIO ABCD), Cologne, Germany; Department of Neurooncology, Center for Neurology, University Hospital Bonn, Bonn, Germany; Center of Integrated Oncology Aachen Bonn Cologne Duesseldorf (CIO ABCD), Cologne, Germany; Department of Radiation Oncology, Helios University Hospital Wuppertal, Faculty of Health, Witten/Herdecke University, Wuppertal, Germany; Institute of Neuroscience and Medicine (INM-3, INM-4, INM-11), Forschungszentrum Juelich, Juelich, Germany; Department of Nuclear Medicine, RWTH University Hospital Aachen, RWTH University Aachen, Aachen, Germany; Institute of Neuroscience and Medicine (INM-3, INM-4, INM-11), Forschungszentrum Juelich, Juelich, Germany; Department of Nuclear Medicine, RWTH University Hospital Aachen, RWTH University Aachen, Aachen, Germany; Institute of Neuroscience and Medicine (INM-3, INM-4, INM-11), Forschungszentrum Juelich, Juelich, Germany; Department of Neurology, RWTH University Hospital Aachen, RWTH University Aachen, Aachen, Germany; Juelich-Aachen Research Alliance (JARA), Section JARA-Brain, Juelich, Germany; Institute of Neuroscience and Medicine (INM-3, INM-4, INM-11), Forschungszentrum Juelich, Juelich, Germany; Center of Integrated Oncology Aachen Bonn Cologne Duesseldorf (CIO ABCD), Cologne, Germany; Department of Radiology and Nuclear Medicine, Maastricht University Medical Center, Maastricht, The Netherlands; Department of Nuclear Medicine, RWTH University Hospital Aachen, RWTH University Aachen, Aachen, Germany; Center for Neurosurgery, Department of General Neurosurgery, Faculty of Medicine and University Hospital Cologne, University of Cologne, Cologne, Germany; Center of Integrated Oncology Aachen Bonn Cologne Duesseldorf (CIO ABCD), Cologne, Germany; Institute of Neuroscience and Medicine (INM-3, INM-4, INM-11), Forschungszentrum Juelich, Juelich, Germany; Institute of Neuroscience and Medicine (INM-3, INM-4, INM-11), Forschungszentrum Juelich, Juelich, Germany; Department for Stereotaxy and Functional Neurosurgery, Center for Neurosurgery, Faculty of Medicine and University Hospital Cologne, Cologne, Germany; Center of Integrated Oncology Aachen Bonn Cologne Duesseldorf (CIO ABCD), Cologne, Germany; Department of Neurology, Faculty of Medicine and University Hospital Cologne, University of Cologne, Cologne, Germany; Institute of Neuroscience and Medicine (INM-3, INM-4, INM-11), Forschungszentrum Juelich, Juelich, Germany

**Keywords:** amino acid PET, BOLD signal, cancer neuroscience, functional MRI, prognostic biomarker

## Abstract

**Background:**

Amino acid PET using the tracer *O*-(2-[^18^F]fluoroethyl)-l-tyrosine (FET) is one of the most reliable imaging methods for detecting glioma recurrence. Here, we hypothesized that functional MR connectivity between the metabolic active recurrent tumor region and resting-state networks of the brain could serve as a prognostic imaging biomarker for overall survival (OS).

**Methods:**

The study included 82 patients (26–81 years; median Eastern Cooperative Oncology Group performance score, 0) with recurrent gliomas following therapy (WHO-CNS 2021 grade 4 glioblastoma, *n* = 57; grade 3 or 4 astrocytoma, *n* = 12; grade 2 or 3 oligodendroglioma, *n* = 13) diagnosed by FET PET simultaneously acquired with functional resting-state MR. Functional connectivity (FC) was assessed between tumor regions and 7 canonical resting-state networks.

**Results:**

WHO tumor grade and IDH mutation status were strong predictors of OS after recurrence (*P* < .001). Overall FC between tumor regions and networks was highest in oligodendrogliomas and was inversely related to tumor grade (*P* = .031). FC between the tumor region and the dorsal attention network was associated with longer OS (HR, 0.88; 95%CI, 0.80–0.97; *P* = .007), and showed an independent association with OS (HR, 0.90; 95%CI, 0.81–0.99; *P* = .033) in a model including clinical factors, tumor volume and MGMT. In the glioblastoma subgroup, tumor volume and FC between the tumor and the visual network (HR, 0.90; 95%CI, 0.82–0.99, *P* = .031) were independent predictors of survival.

**Conclusions:**

Recurrent gliomas exhibit significant FC to resting-state networks of the brain. Besides tumor type and grade, high FC between the tumor and distinct networks could serve as independent prognostic factors for improved OS in these patients.

## Graphical Abstract

**Graphical Abstract:**
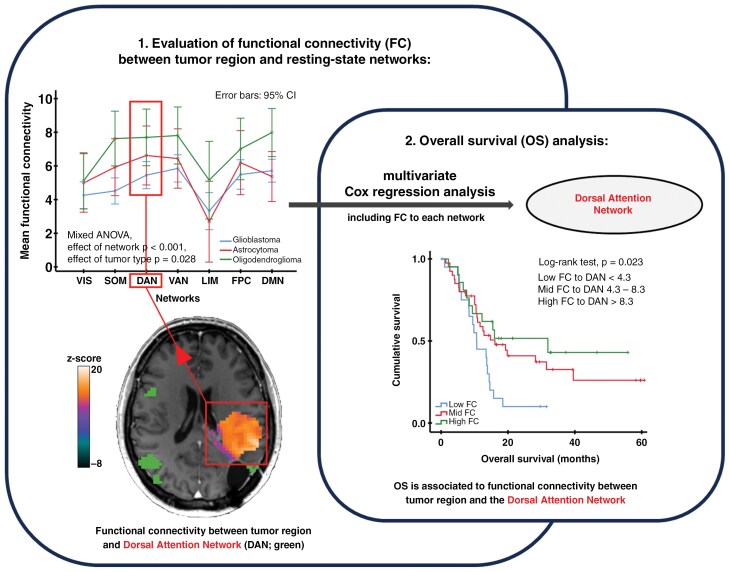

**Importance of the Study**

There is increasing evidence in oncological neuroscience that preserved functional connectivity (FC) between tumor-infiltrated brain regions and resting-state networks of the brain influence overall survival in primary gliomas. Here, we found that FC between the brain region infiltrated by metabolically active tumors and different resting-state networks also prevails in recurrent gliomas of different types and grades and is associated with overall survival. These results suggest that FC can be used as a novel prognostic biomarker for imaging in patients with recurrent glioma.

**Key Points**

- Recurrent gliomas exhibit functional connectivity to resting-state networks.
- Functional connectivity is higher in IDH-mutant gliomas than in glioblastomas.
- Tumor connectivity to visual/attention networks is a factor for survival.

Gliomas are primary brain tumors with a strong tendency to infiltrate the normal brain tissue diffusely.^1^ This growth pattern leads to a close interaction between tumor cells and the local microenvironment that comprises neurons, normal glia, and immunogenic and inflammatory cells.^2^ In particular, the interaction between neuronal elements and glioma cells has recently attracted considerable attention and is now considered a critical aspect in the emerging field of *Cancer Neuroscience*.^3,4^

From a neurooncological perspective, the impact of neuronal activity on glial cells is an important issue, and there is clear evidence that neuronal activity induces and promotes glioma growth through paracrine signaling and the formation of excitatory glutaminergic synapses between neurons and glioma cells.^5–8^ Conversely, developing a macroscopic glioma may affect neuronal activity and signal conduction locally and at distant sites. The functional impact of local glioma infiltration into the adjacent cortex has mainly been studied in the context of presurgical mapping before tumor resection. Typical task-evoked patterns of neuronal activity, assessed using electrophysiological methods^9–13^ or magnetencephalography,^13,14^ may be preserved in the glioma-infiltrated cortex.

Besides local effects, glioma growth and invasion may affect the integrity of whole-brain neural networks. Resting-state functional magnetic resonance imaging (rs-fMRI) has been used to characterize alterations in whole-brain connectivity in patients with glioma, with consistent evidence of disturbed functional connectivity (FC) extending to the contralateral hemisphere.^15,16^ Moreover, global network connectivity changes may serve as imaging biomarkers predicting survival predominantly in glioblastomas.^17–20^ In addition, FC between tumor-infiltrated regions and networks of the brain has been investigated in newly diagnosed glioblastoma^21^ and mixed cohorts of patients with primary and recurrent CNS WHO grade 2–4 gliomas.^22^ In these studies, tumor voxels were frequently functionally connected to resting-state networks^21^ or otherwise identified cortical areas,^22^ and preserved FC was associated with better overall survival in certain subgroups.

In recurrent gliomas, the interactions of different therapeutic interventions comprising tumor resection, radiotherapy, and chemotherapy using alkylating drugs with glioma cells, neurons, and immunogenic/inflammatory cells may further complicate the situation, rendering outcome and survival less predictable^23–25^

In the present study, we investigated the FC between brain regions infiltrated by metabolic active tumors and resting-state networks using rs-fMRI in patients with recurrent glioma, including its prognostic value. In addition to anatomical MRI, diagnosis, localization, and extent of recurrent gliomas were assessed using amino acid PET with the tracer *O*-(2-[^18^F]fluoroethyl)-l-tyrosine (FET), one of the most reliable noninvasive imaging methods for detecting glioma recurrence.^26^

## Patients and Methods

### Patient Characteristics

Patients were recruited as part of a prospective study that included glioma patients with a suspected recurrence/progression who were in good general condition (ECOG performance score, 0–1 at screening), had no major depression, were free of seizures (with or without anticonvulsive medication), and were able to undergo anatomical MRI, functional rs-fMRI and FET PET acquired simultaneously on a hybrid MR/PET scanner (for imaging protocols, see Supplementary Material). The Ethics Committee of the University of Cologne approved the study protocol (protocol number 17-365), and written informed consent was obtained from all patients per the Declaration of Helsinki.

Eighty-two adults (*n* = 36 women; *n* = 46 men; median age, 53 years; range, 26–81 years) with gliomas at recurrence were included. All tumors were histomolecularly characterized according to the 2021 classification of the WHO for Tumors of the CNS.^27^ Patients were referred to the imaging facility of the Forschungszentrum Juelich (Research Center Juelich), Germany, by 5 university hospitals between February 2018 and August 2022. All patients had *Progressive Disease* on anatomical MRI according to the RANO 1.0 criteria^28^ and a metabolically active tumor with pathologically increased tracer uptake as assessed by FET PET. The criteria for the additional FET PET-based diagnosis of glioma recurrence have been described previously.^29^

Fifty-seven patients (70%) had a CNS WHO grade 4 glioblastoma, 12 patients (14%) had a CNS WHO grade 3 or 4 astrocytoma, and 13 patients (16%) had a CNS WHO grade 2 or 3 oligodendroglioma (Table 1). Overall, 57 (70%) patients had IDH-wild-type tumors, and 25 (30%) patients had IDH-mutant tumors. First-line therapy had been applied according to respective guidelines at the initial diagnosis. For instance, most of the glioblastomas (83%) had undergone first-line therapy comprising surgery or biopsy, postoperative radiotherapy with concomitant and maintenance temozolomide chemotherapy,^30^ or temozolomide plus lomustine chemoradiation according to the CeTeG/NOA-09 trial.^31^ In patients with astrocytomas, a combination of surgery or biopsy, radiotherapy with concomitant and maintenance temozolomide chemotherapy was the most common first-line therapy (83%). At initial diagnosis, patients with oligodendroglioma had undergone either surgery alone or surgery followed by radiotherapy and adjuvant nitrosourea-based chemotherapy.

**Table 1.** Patient Characteristics

	*N*	% or range
Sex (male/female)	46/36	56/44%
Median age and range (years)	53	26 - 81
ECOG score (0/1/2/3)	48/28/5/1	59/34/6/1%
Main symptom		
None	34	41%
Aphasia	10	12%
Paresis	17	21%
Fatigue, Dizziness	12	15%
Vision Impairment	9	11%
Tumor type and WHO CNS tumor grade		
Glioblastoma, IDH-wild-type, CNS WHO grade 4	57	69%
Astrocytoma, IDH-mutant, CNS WHO grade 3/4	9/3	11/4%
Oligodendroglioma, IDH-mutant, CNS WHO grade 2/3	10/3	12/4%
IDH mutational status		
Wild-type/mutant	57/25	69/31%
MGMT promoter methylation status		
Methylated/non-methylated/unknown	47/26/9	57/32/11%
FET PET tumor location^a^		
Frontal left/right	16/15	20/18%
Parietal left/right	6/13	7/16%
Temporal left/right	13/14	16/17%
Occipital left/right	2/2	2/2%
Basal ganglia left	1	1%
Lesion volumes (mL)		
Resection cavity	3.2	0.0 - 124.0
FLAIR hyperintensity	59.4	8.0 - 250.9
T1 contrast-enhancing lesions	5.6	0.0 - 80.5
FET PET (TBR > 1.6)	36.8	2.4 - 226.3
Interval between initial diagnosis and recurrence (months)		
Glioblastoma	9.4	0.4 - 97.5
Astrocytoma	44.6	0.9 - 80.8
Oligodendroglioma	59.3	1.3 - 145.4
Pretreatment (number of procedures)		
Surgery^b^ (1/2/3/4)	69/10/2/1	84/12/3/1%
Radiotherapy (0/1/2)	9/65/8	11/79/10%
Chemotherapy (0/1/2/3/4)	14/51/12/3/2	17/62/15/4/2%
Extent of resection at initial diagnosis		
None/biopsy/partial/complete	3/18/9/52	4/22/11/63%
Corticosteroids (no/yes)	63/19	77/23%
Anticonvulsants (no/yes)	27/55	33/67%

Abbreviations: ECOG, Eastern Cooperative Oncology Group performance score; IDH, isocitrate dehydrogenase; MGMT, O^6^-methylguanine-DNA methyltransferase; FLAIR, fluid-attenuated inversion recovery; FET, O-(2-[^18^F]fluoroethyl)-L-tyrosine; TBR, tumor-to-brain ratio; ^a^main lobe involved; ^b^including biopsy.

The median time interval between initial diagnosis and recurrence depended on tumor type (glioblastoma, 9.4 months; astrocytoma, 44.6 months; oligodendroglioma, 59.3 months). Table 1 shows the number of therapy lines (eg, surgical interventions including stereotactic biopsy, radiotherapy series, and chemotherapies) applied until the time of diagnosis of recurrence.

### Image Preprocessing and Generation of Lesion Masks

The functional imaging data passed the standard SPM12/CONN toolbox preprocessing steps.^32^ These include motion correction, removal of outliers, regressing out noise signals from the cerebrospinal fluid and white matter, slice-timing correction, smoothing with 5 mm FWHM, and bandpass filtering to 0.008–0.09 Hz. Afterward, functional and structural images were non-rigidly co-registered to the MNI-152 standard brain template using the SPM12/CONN unified segmentation and registration algorithm. From the structural MR and PET images, 4 binary lesion masks were generated for the subsequent analysis of FC. These included lesions with pathologically increased FET uptake, T1 contrast-enhancing lesions, T2/FLAIR hyperintensities, and resection cavities. Segmentation of tumor regions with pathologically increased FET PET uptake at a tumor-to-brain ratio of 1.6 or more^33^ was performed using an FSL script (FSL toolbox, https://fsl.fmrib.ox.ac.uk). For this purpose, the PET images were converted into standardized tumor-to-brain ratio images whereby the average tracer activity in the unaffected brain served as the reference. T1 contrast-enhancing lesions and T2/FLAIR hyperintensities were automatically segmented using the deep learning software HD-GLIO (https://github.com/NeuroAI-HD/HD-GLIO), while resection cavities were manually contoured by an experienced radiation oncologist (MK). All segmentations were visually inspected, manually corrected, and finally merged into a composite lesion mask. The location of the metabolic active, FET-avid recurrent tumors with respect to the 4 major lobes of each hemisphere was calculated from the maximal volumetric overlap of the PET mask with the lobe definitions from the MNI-152 standard brain template.

### Determination of FC Between Tumor Region and Networks

FC between the metabolically active tumor region and a set of specified resting-state networks was determined by a variant of the FSL dual regression method^34–36^ where atlas-based network masks served as seeds. We analyzed 7 resting-state networks defined in the Yeo atlas,^37^ ie, the visual, somatomotor, dorsal attention, ventral attention, limbic, fronto-parietal control, and default mode networks. The corresponding binary atlas network masks were cropped by the individual pathological areas (composite lesion masks) and served as seed regions. The average blood-oxygen-level-dependent (BOLD) time series from the voxels in the cropped network masks was calculated and used as a regressor. Network-specific regression coefficients were then calculated for all voxels, normalized to z-scores, and stored as connectivity maps describing the strength of FC between each voxel and the analyzed network. Finally, we computed the average normalized regression coefficient of the voxels within the segmented PET lesion to obtain the FC between the tumor region and each of the 7 networks. The average of the individual network connectivity values was used as a measure of whole-brain connectivity. In addition, the spatial proximity of the tumor to the resting-state networks was determined where we used the center of gravity of the PET lesion and the centroids of the network nodes to calculate the mean distance (proximity) between the PET lesion and each network.

### Statistical Analysis

Statistical analysis was performed using the SPSS statistical software package (version 25, IBM Corporation). Independent-sample two-sided *t*-tests or one-way ANOVA were performed to determine group differences for continuous variates. A mixed, repeated-measures ANOVA with Greenhouse-Geisser correction for non-sphericity was used to analyze differences in FC between individual networks and as a function of tumor type, IDH mutational status, and CNS WHO tumor grade. For analysis of overall survival, the duration between the date of imaging and the date of death was recorded, or data were censored at the time of the last available follow-up. The association of FC between brain regions infiltrated by metabolic active tumor and networks alone as well as along with tumor-related (tumor type, CNS WHO tumor grade, IDH mutational status) and other common prognostic factors (ie, age, the extent of resection, Eastern Cooperative Oncology Group (ECOG) performance score, O^6^-methylguanine-DNA methyltransferase (MGMT) promotor methylation status, metabolic active tumor volume) on overall survival was estimated using Kaplan–Maier analysis with log-rank tests or Cox regression analysis. For Kaplan–Maier analysis of FC, patients were grouped into quartiles or with respect to the median. For the multivariate survival analysis, either a Cox regression analysis with simultaneous or stepwise-forward inclusion of variables was used (detailed in *Results*). Forward inclusion was additionally validated by stepwise backward exclusion. Unless otherwise stated, *P*-values less than .05 were considered statistically significant. Survival analysis was also performed in the subgroup of IDH-wild-type glioblastoma patients.

## Results

**Figure 1.**
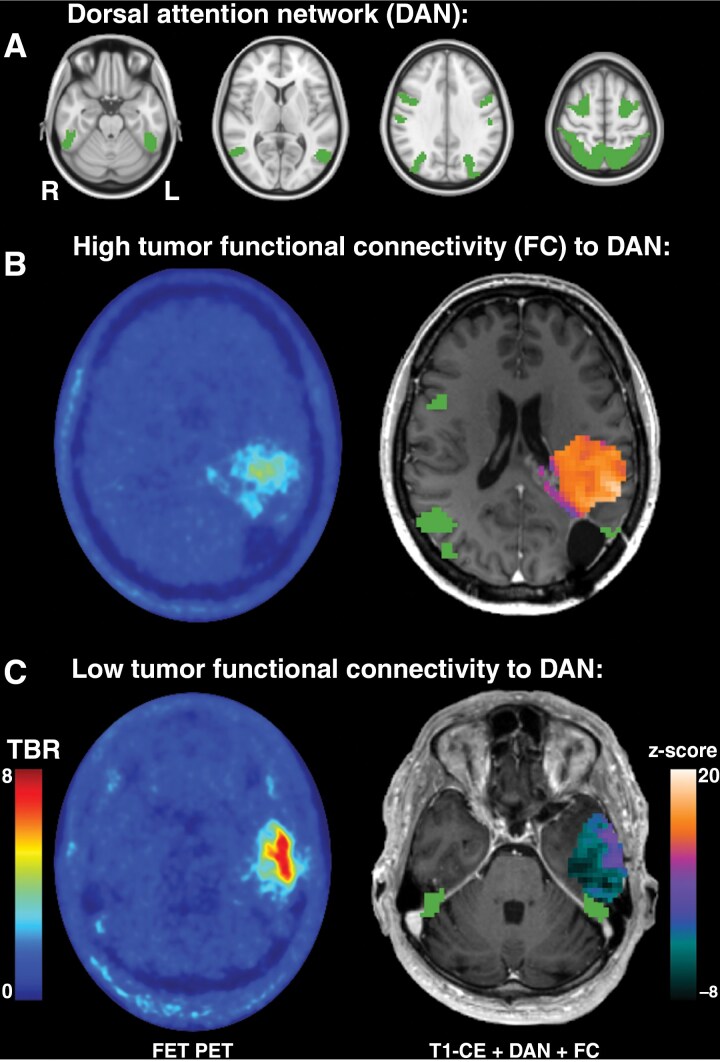
(A) Location of the dorsal attention network (DAN) in the MNI-152 standard space. (B) and (C), exemplary patients with high or low functional connectivity between brain regions infiltrated by metabolic active tumor and the DAN (right) and the corresponding *O*-(2-[^18^F]fluoroethyl)-l-tyrosine (FET) PET images (left). Functional connectivity (FC) of the tumor region was superimposed on the T1-weighted contrast-enhanced (T1-CE) image together with the DAN mask from (A). Abbreviation: TBR, tumor-to-brain-ratio.

Most recurrent tumors were in the frontal (*n* = 31; 39%) or temporal (*n* = 27; 33%) lobes. The median volume of the FET PET-avid lesions was 36.8 mL (range, 2.4–226.3 mL), and the median average tumor-to-brain ratio (TBR_mean_) was 2.17 (range, 1.88–2.94). Metabolic active tumor volume did not depend on the tumor type, CNS WHO tumor grade, or IDH mutational status (ANOVA and two-sided *t*-test, *P* > .05). In contrast, tumor-to-brain ratios depended on the CNS WHO tumor grade (ANOVA, *P* = .031) but not on the tumor type or IDH mutational status (ANOVA and two-sided *t*-test, *P* > .05). Figure 1 shows FET PET images of two exemplary patients.

### FC Between Tumor Region and Resting-State Networks

**Figure 2.**
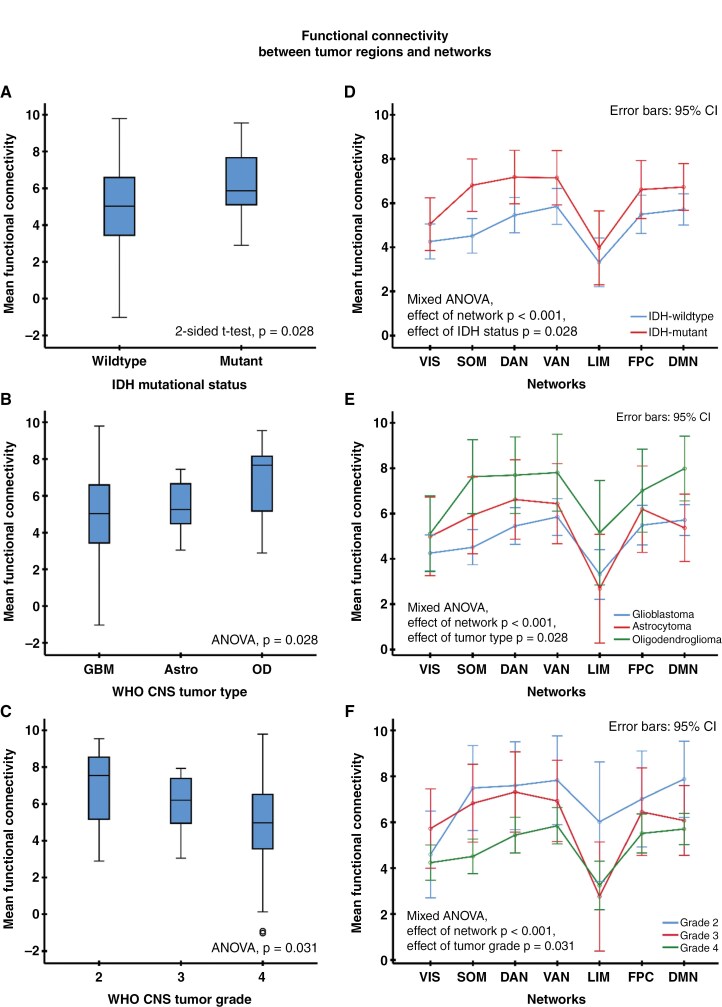
Mean overall functional connectivity between brain regions infiltrated by metabolic active tumor and 7 canonical resting-state networks (A-C) as well as network-specific connectivity (D-F) in patients with recurrent glioma related to the IDH mutational status (top row), tumor type (middle row), and CNS WHO tumor grade (bottom row). Abbreviations: IDH, isocitrate dehydrogenase; ANOVA, analysis of variance; CI, confidence interval; VIS, visual; SOM, somatomotor network; DAN, dorsal attention network; VAN, ventral attention network; LIM, limbic network; FPC, frontal-parietal control network; DMN, default mode network; GBM, glioblastoma; Astro, astrocytoma; OD, oligodendroglioma

Overall FC between brain regions infiltrated by metabolic active tumor and resting-state networks (mean of all 7 canonical networks) was significantly higher in IDH-mutant gliomas than in IDH-wild-type gliomas (two-sided *t*-test, *P* = .028; Figure 2A). Also, it depended significantly on the WHO CNS tumor type (ANOVA, *P* = .028; Figure 2B). In IDH-mutant gliomas, oligodendrogliomas had the highest FC (z-score, 6.9 ± 1.9), while in astrocytomas the FC was closer to that of the glioblastomas (z-score, 5.5 ± 1.3 vs. 4.9 ± 2.6; Figure 2B). In addition, connectivity was inversely related to the CNS WHO tumor grade (ANOVA, *P* = .031; Figure 2C). FC of the PET-avid tumor regions differed significantly concerning the 7 resting-state networks (mixed repeated-measures ANOVA, within subjects’ effect, *P* < .001; Figure 2D-F). Overall, the highest connectivity was observed between the tumor regions and the ventral attention (z-score, 6.2 ± 3.1), dorsal attention (z-score, 6.0 ± 3.1), default mode (z-score, 6.0 ± 2.7) and frontoparietal control (z-score, 5.8 ± 3.3) networks, while the lowest connectivity was to the limbic network (z-score, 3.5 ± 4.2). Furthermore, the observed association between tumor type, CNS WHO tumor grade, IDH mutational status, and whole-brain connectivity was well preserved throughout all networks (mixed ANOVA, between subjects’ effect, range of *P*-values, .028–.031; Figure 2D-F). Of note, the connectivity between the tumor region and networks was not associated with the tumor location regarding the cerebral lobes (ANOVA, *P* = .96). In addition, the FC was inversely related to the proximity of the tumor to the resting-state networks (*P* < .05 for most networks, see Supplementary Table S1 and Figure S1).

### Association of FC Between Tumor Region and Resting-State Networks and Overall Survival

**Figure 3.**
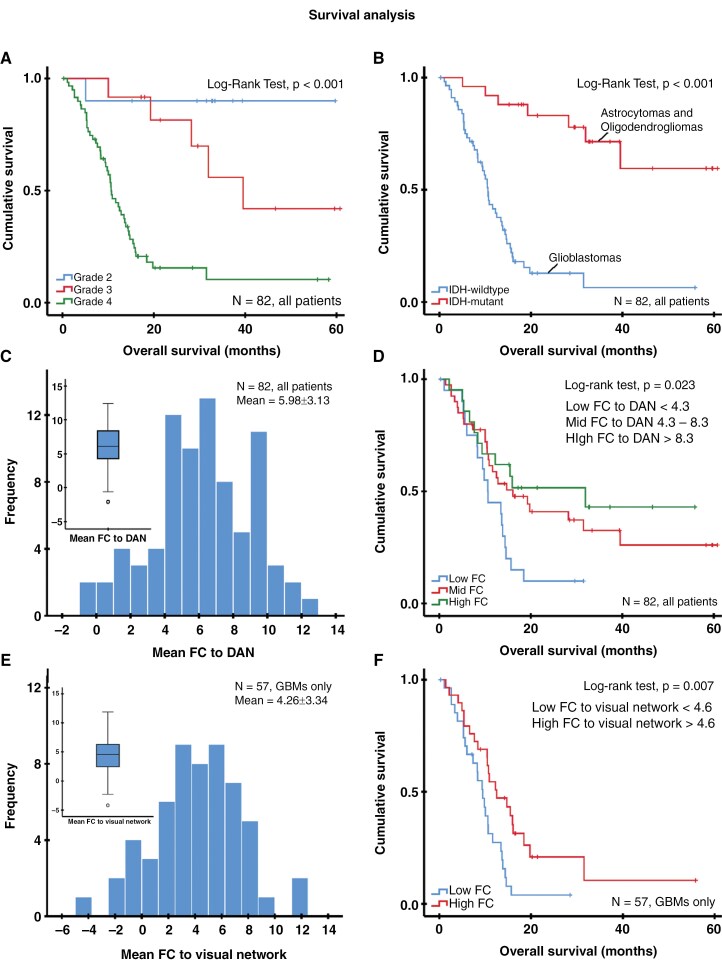
Kaplan–Meier survival curves in patients with recurrent gliomas of different CNS WHO tumor grades (A), different IDH mutational status (B), and various levels of functional connectivity (low, middle, and upper quartile) between brain regions infiltrated by metabolic active tumor and the dorsal attention network (D). The distribution of network connectivity between the tumor regions and the dorsal attention network is visualized as a histogram and boxplot (C). Results for the IDH wild-type glioblastoma subgroup regarding the distribution of network connectivity between tumor and visual network are shown in (E), as well as Kaplan–Meier survival curves with a median (4.6) classification of connectivity into high and low in (F). Abbreviations: IDH, isocitrate dehydrogenase; FC, functional connectivity; DAN, dorsal attention network; N, number of patients.

The overall survival after diagnosis of tumor recurrence diagnosed by FET PET was significantly associated with the tumor type, CNS WHO grade, and IDH mutational status (Kaplan–Meier analysis, all *P* < .001; Figure 3A, 3B). Univariate Cox regression analysis revealed a significant association between improved overall survival and the connectivity of brain regions infiltrated by metabolic active tumor with the visual, somatomotor, and DAN (range of *P*-values, .041–.007; Table 2). Subsequent stepwise forward and confirmatory backward multivariate Cox regression analysis including all 7 networks refined this association to the dorsal attention network (DAN) alone (HR, 0.88; 95% CI, 0.80–0.97; *P* = .007, Table 2). Figure 1 shows the DAN and two exemplary patients with high and low FC between the tumor region and the DAN. The connectivity between the tumor region and the DAN was almost normally distributed (z-score, 5.98 ± 3.13; Figure 3C). Patients with the highest FC between tumor and DAN (upper quartile, z-score > 8.3, *n* = 21) had a median overall survival of 31.9 months. In contrast, patients with intermediate (middle quartiles, z-score 4.3–8.3, *n* = 40) or low FC (lower quartile, z-score < 4.3, *n* = 21) had a median overall survival of 16.1 months and 10.6 months, respectively (log-rank test, *P* = .023; Figure 3D).

**Table 2.** Univariate and Multivariate^#^ Cox Regression Analysis for Association Between Tumor-to-Network Resting-State Functional Connectivity and Overall Survival in Recurrent Gliomas

	Entire patient cohort		Glioblastoma patients	
Resting-state network	*P*-value (univariate)	*P*-value (multivariate)^#^	*P*-value (univariate)	*P*-value (multivariate)^#^
Visual	.036	.677	.023	.023
Somatomotor	.041	.720	.794	.329
Dorsal Attention	.007	.007	.107	.936
Ventral Attention	.246	.101	.875	.222
Limbic	.997	.900	.759	.838
Frontoparietal	.309	.159	.704	.370
Default Mode	.453	.133	.946	.102

^#^forward stepwise inclusion.

Furthermore, FC between the metabolic active tumor regions and the DAN had an independent association with overall survival (HR, 0.90; 95% CI, 0.81–0.99; *P* = .033) in a multivariate model (Table 3) that excluded features correlated with connectivity (IDH, tumor type, tumor grade) but included other tumor-related variables such as the metabolically active tumor volume (*P* = .023) and MGMT promoter methylation status (*P* = .057), as well as common prognostic factors such as age (*P* = .215), extent of resection at initial diagnosis (*P* = .162), and ECOG score (*P* = .172).

**Table 3.** Univariate and Multivariate Cox Regression Analysis to Evaluate Tumor-Related and Common Prognostic Factors on Overall Survival in Recurrent Gliomas Diagnosed With FET PET

	Entire patient cohort, FC between tumor and dorsal attention network		GBM patients, FC between tumor and visual network	
Factor	*P*-value (univariate)	*P*-value (multivariate)	*P*-value (univariate)	*P*-value (multivariate)
Connectivity between tumor and network	.007	.033	.023	.031
Metabolic active tumor volume	.012	.023	.003	.011
Age	.237	.215	.887	.232
MGMT promoter methylation status	.003	.057	.319	.314
Extent of resection at initial diagnosis	.008	.162	.961	.824
ECOG score	.001	.172	.051	.865

In the survival analysis of the subgroup of IDH-wild-type glioblastoma patients, univariate and subsequent stepwise forward and confirmatory backward multivariate Cox regression analysis comprising all 7 networks indicated a significant association between improved overall survival and the connectivity of the metabolic active tumor regions with the visual network (*P*-values, univariate and multivariate .023; Table 2). The connectivity with the visual network was almost normally distributed (z-score, 4.26 ± 3.34; Figure 3E). Overall survival of the glioblastoma patients differed significantly depending on the level of FC between tumor region and visual network (Kaplan–Maier analysis, log-rank test, *P* = .007; Figure 3F), where patients with high FC (z-score > 4.6 [median], *n* = 29) had a median overall survival of 12.5 months and patients with low FC (z-score < 4.6 [median], *n* = 28) one of 9.5 months. A final multivariate model including metabolically active tumor volume (*P* = .011), MGMT promoter methylation status (*P* = .314), age (*P* = .232), extent of resection at initial diagnosis (*P* = .824) and ECOG score (*P* = .865) confirmed the independent association of FC between tumor region and the visual network with overall survival (HR, 0.90; 95% CI, 0.82–0.99; *P* = .031; Table 3). The mean distances (proximities) of the PET lesions to the DAN (all patients, *P* = .34) and to the visual network (IDH-wild-type patients, *P* = .47) were not significantly associated with overall survival in the univariate analyses and were therefore not included in the multivariate models.

## Discussion

### Main Findings

The present study evaluated FC between the brain region infiltrated by metabolic active tumor and a set of canonical resting-state networks using the BOLD signal of the metabolic active tumor region in a cohort of patients with recurrent gliomas of varying histomorphologic and molecular characteristics. Tumor regions were diagnosed by a combination of anatomical MRI and FET PET, and FC of the metabolic active tumor differed between the networks and was highest with the 4 large associative networks (ie, the dorsal attention, ventral attention, frontoparietal control, and default mode network). Overall and network-specific connectivity of tumor regions was highest in oligodendroglioma and was significantly higher in IDH-mutant than in IDH-wild-type tumors. In the overall cohort, CNS WHO tumor grade and IDH mutational status were the most significant prognostic factors for longer overall survival but preserved FC between recurrent glioma and the DAN was also a prognostic factor. Importantly, in the subgroup of recurrent IDH-wild-type glioblastoma, connectivity between the tumor region and the visual network was an independent predictor of overall survival. The present investigation differs from similar recent reports^11,13,21,22^ in that FET PET was used for tumor definition, that FC of gliomas of different WHO CNS types and grades was systematically analyzed, and that only patients with recurrent gliomas were included. FET PET was used because it is a highly reliable measure of glioma infiltration. Several biopsy-controlled studies have shown that FET PET in conjunction with an empirical threshold for tumor-to-brain ratio outperformed MRI-based methods in terms of accuracy.^38–40^

### Glioma Interaction Within Directly Infiltrated Cortex

From a clinical point of view, glioma infiltration of normal brain tissue raises the question of whether the tumor-infiltrated cortex is still functional and can be safely resected. Classical electrophysiological mapping techniques comprising direct cortical stimulation and somatosensory evoked potentials have proven the presence of functional cortex within grossly abnormal tumor tissue.^9,10^ These findings were confirmed using advanced electrophysiological methods, such as task-related spectral power perturbations in the high-gamma range^11–13^ and magnetoencephalography.^14^ While these clinical studies do not allow for unraveling details and directions of interactions between glioma cells and neurons, ample experimental evidence shows that neurons connect to glioma cells via electrically active gap junctions and glutaminergic synapses.^8^ In turn, as Krishna et al. outlined,^13^ preclinical studies suggest that glioma cells induce neuronal hyperexcitability in the tumor-infiltrated cortex by glutamate release^41,42^ and reduction of GABAergic inhibitory interneurons.^43^ In patients, neuronal hyperexcitability has also been observed in the form of increased task-induced high-gamma power during intraoperative electrocorticography.^13^ Of note, the interaction between glioma and neurons went far beyond the directly infiltrated cortex, as tasks recruited neighboring cortical areas that were not normally involved.^13^

### Connectivity Between Tumor Region and Brain-Wide Networks

Apart from close local structural and functional connections between glioma tissue and neural elements as characterized above, glioma-infiltrated cortical regions maintain long-range functional connections to other brain regions and networks. For example, Krishna et al. investigated 19 patients with glioblastoma using magnetoencephalography and observed that up to 50% of the intratumoral voxels included in the contrast-enhanced or FLAIR-hyperintense tumor regions were strongly connected to the rest of the brain.^13^ Furthermore, Mandal and co-workers^11^ used rs-fMRI in 17 low-grade gliomas to evaluate the participation of tumor-infiltrated cortex in large-scale cognitive circuits, using the same network definition as in the present study.^37^ Of note, the tumor locations that were active during a task that probed executive functions were found to exhibit significant FC with the DAN. More recently, Daniel and colleagues^21^ evaluated 57 patients with newly diagnosed glioblastoma using rs-fMRI. By applying a heuristic threshold, up to 60% of intratumoral voxels were functionally connected to at least 1 of the 7 canonical networks, although this proportion largely varied between patients. In addition, tumor voxels did not connect specifically to any resting-state network. Another study evaluated FC between tumor and brain in 54 patients with CNS WHO grade 2–4 gliomas in the newly diagnosed or recurrent setting.^22^ Again, significant resting-state FC between the tumors and the unaffected brain was observed in that study. In the newly diagnosed tumors, the set of brain voxels functionally connected with the tumor resembled the frontoparietal control network, while in the recurrent tumors, the location of the connected brain voxels resembled the DAN.^22^

Regarding glioma grade and IDH mutational status, it can be expected that the lower the tumor grade and the less aggressive the tumor growth, the higher the preserved neuronal population and the less disturbed the local and distant functional organization of the infiltrated cortex, as observed here in recurrent gliomas. Taken together, these results, including the present study’s findings, suggest that in both newly diagnosed and recurrent gliomas, a substantial proportion of cortical tissue infiltrated by the glioma is locally functional and remains remotely connected. FC between the brain region infiltrated by metabolic active tumor and other parts of the brain is present to varying degrees in all canonical networks and does depend on the location but rather on the tumor’s invasiveness.

### Association Between Tumor Region and Brain Connectivity and Survival

In the present study, we observed a significant association of FC between the region infiltrated by metabolic active tumor and the brain and overall survival. In this sense, a higher FC between the tumor region and the visual, somatomotor, and DAN was associated with a lower risk for death. Similarly, Daniel et al.^21^ found that higher tumor intra-network connectivity was associated with longer overall survival in patients with newly diagnosed glioblastoma. Interestingly, reduced or absent FC was observed in the necrotic tumor regions compared to the solid, contrast-enhancing regions. The authors hypothesized that tumors with better-preserved physiology have a more favorable prognosis, which is supported by the observation in the present study that FC between tumor regions and networks was higher in CNS WHO grade 2 or 3 and IDH-mutant gliomas than in IDH-wild-type gliomas. Nevertheless, these results are only partly in line with those from Sprugnoli et al.^22^ in newly diagnosed gliomas, where the connectivity of the solid tumor with various contiguous brain regions correlated either positively (right parieto-temporal) or negatively (right or left frontal) with the duration of survival. However, in recurrent malignant gliomas, only one positively correlated cluster (i.e. right frontal) was identified.^22^ These partly discrepant findings may result from the varying methods of defining the solid parts in tumors of different tumor grades, which we here attempted to overcome by adding amino acid PET with the tracer FET to anatomical MRI in all examined patients.

The present observation that the connectivity between brain regions infiltrated by metabolic active tumor and the DAN (whole cohort) and the visual network (IDH-wild-type glioblastoma) was associated with a significant improvement in overall survival could be related to the specific function of this networks for cognitive performance. The DAN is mainly involved in goal-directed, voluntary control of visuospatial attention,^44,45^ a cognitive domain that plays an important role for executive functioning and is naturally also depending on the visual network. In addition, the visual network is an important resource not only for object recognition but also for spatial orientation and goal-directed actions with objects^46^ and for posture^47^ and thus has a major influence on daily physical functioning. As mentioned, Mandal and colleagues^11^ observed that tumor-infiltrated cortical regions that were active during an executive task may have significant FC with the DAN. Although not specifically addressing the DAN, Krishna, and colleagues^13^ also observed that gliomas may remodel FC such that task-relevant neural responses activate tumor-infiltrated cortex well beyond the cortical regions that are normally recruited in the healthy brain. Thus, the improved survival of IDH-mutant or wild-type gliomas could in part be related to the ability of the affected brain to recruit tumor-infiltrated, distant regions to serve vision, visual attention, and executive functions which are closely associated with prognosis.^48^

### Limitations

Only a limited number of patients were available for the present study, and these showed some heterogeneity in treatment. Therefore, important relationships between FC and overall survival may have been overlooked in the analysis, especially in the group of IDH-mutant gliomas.

## Conclusion

The present results indicate that recurrent gliomas, as defined by pathologically increased FET uptake, maintain functionally connected to most of the major known resting-state networks. FC between brain regions infiltrated by metabolic active tumors and networks was generally higher in low-grade and IDH-mutant recurrent gliomas, and connectivity with the dorsal attention and visual networks proved to be an additional prognostic factor for improved overall survival. Thus, the close glioma-neural relationships observed in primary gliomas appear also to play a prognostic role in recurrent gliomas, suggesting that FC may be used as a novel prognostic imaging biomarker in patients with recurrent gliomas.

## Supplementary Material

vdaf023_suppl_Supplementary_Material

## Data Availability

Anonymized and aggregated data are available on reasonable request.
