# Supplementary material for: Functional connectivity between tumor region and resting-state networks as imaging biomarker for overall survival in recurrent gliomas diagnosed by O-(2-[18F]fluoroethyl)-l-tyrosine PET
Source: Neurooncol Adv. 2025 Jan 29;7(1):vdaf023. doi: 10.1093/noajnl/vdaf023 (PMC11904474; doi:10.1093/noajnl/vdaf023)
Supplement: vdaf023_suppl_Supplementary_Material [file vdaf023_suppl_supplementary_material.docx]

**Supplemental Material**

**Functional connectivity between tumor region and resting-state networks as imaging biomarker for overall survival in recurrent gliomas diagnosed by FET PET**

Michel Friedrich, Jan-Michael Werner, Joachim P. Steinbach, Michael Sabel, Ulrich Herrlinger, Marc Piroth, Gabriele Stoffels, Christian P. Filss, Philipp Lohmann, Nadim J. Shah, Maximilian I. Ruge, Felix M. Mottaghy, Roland Goldbrunner, Karl-Josef Langen, Gereon R. Fink, Martin Kocher*, and Norbert Galldiks*

***equal contribution to senior authorship**

*Institute of Neuroscience and Medicine (INM-3, INM-4, INM-11), Forschungszentrum Juelich, Juelich, Germany (MF, GS, CPF, PL, NJS, KJL, GRF, MK and NG)*

*Department of Neurology, Faculty of Medicine and University Hospital Cologne, University of Cologne, Cologne, Germany (JMW, GRF and NG)*

*Center of Integrated Oncology Aachen Bonn Cologne Duesseldorf (CIO ABCD), Germany (MS, UH, MIR, RG, MK and NG)*

*Department of Neuro-Oncology, University Hospital Frankfurt - Goethe-University, Frankfurt, Germany (JPS)*

*Department of Neurosurgery, Heinrich Heine University Medical Faculty and University Hospital Düsseldorf, Düsseldorf, Germany (MS)*

*Department of Neurooncology, Center for Neurology, University Hospital Bonn, Bonn, Germany (UH)*

*Department of Radiation Oncology, Helios University Hospital Wuppertal, Faculty of Health, Witten/Herdecke University, Wuppertal, Germany (MP)*

*Juelich-Aachen Research Alliance (JARA), Section JARA-Brain, Juelich, Germany (NJS)*

*Department of Neurology, RWTH University Hospital Aachen, RWTH University Aachen, Aachen, Germany (NJS)*

*Department for Stereotaxy and Functional Neurosurgery, Center for Neurosurgery, Faculty of Medicine and University Hospital Cologne, Cologne, Germany (MIR and MK)*

*Department of Nuclear Medicine, RWTH University Hospital Aachen, RWTH University Aachen, Aachen, Germany (CPF, PL, FMM, KJL)*

*Department of Radiology and Nuclear Medicine, Maastricht University Medical Center, Maastricht, Netherlands (FMM)*

*Center for Neurosurgery, Department of General Neurosurgery, Faculty of Medicine and University Hospital Cologne, University of Cologne, Cologne, Germany (RG)*

**Imaging protocols**

*O*-(2-[^18^F]-fluoroethyl)-L-tyrosine (FET) PET, structural MR, and resting-state functional magnetic resonance imaging (rs-fMR) images were acquired simultaneously using a 3T hybrid scanner (Siemens Tim-Trio/BrainPET, Siemens Medical Systems, Erlangen, Germany). For this purpose, the scanner was equipped with a birdcage-like quadrature transmitter head coil mounted on the couch, an 8-channel receiver coil, and a PET insert consisting of 72 rings (axial field-of-view, 19.2 cm; center spatial resolution, 3 mm FWHM). FET PET images were obtained following Herzog et al.^1^.

The structural MRI protocol comprised a 3D high-resolution T1-weighted magnetization-prepared rapid acquisition gradient echo (MPRAGE) native scan (176 slices; repetition time TR = 2250 ms; echo time TE = 3.03 ms; field of view (FoV) = 256 × 256 mm^2^; flip angle = 9°; voxel size = 1 × 1 × 1 mm^3^), a contrast-enhanced MPRAGE scan recorded after injection of gadolinium-based contrast agent, a T2-weighted sampling perfection with application-optimized contrasts using different flip angle evolution scan (176 slices; repetition time TR = 3.2 ms; echo time TE = 417 ms; FoV = 256 × 256 mm^2^; voxel size = 1 × 1 × 1 mm^3^), and a T2-weighted fluid-attenuated inversion recovery (T2/FLAIR) scan (25 slices; TR = 9000 ms; TE = 3.86 ms; FoV = 220 × 220 mm^2^; flip angle = 150°; voxel size = 0.9 × 0.9 × 4 mm^3^). To acquire rs-fMRI data, patients were instructed to relax and let their minds wander, but not fall asleep. Within 11 minutes, a total of 300 functional volumes were acquired with a gradient-echo echo-planar imaging pulse sequence (36 axial slices; slice thickness, 3.1 mm; TR = 2200 ms; TE = 30 ms; flip angle = 90°; FoV = 200 × 200 mm^2^; in-plane voxel-size, 3.1 × 3.1 mm^2^).

**Table S1:** Regression analyses for the entire patient cohort between the mean functional connectivity (z-score) of the metabolically active tumor region and its proximity (mm) to the networks.

| **Resting-State Network** | **Coefficient of Determination (R^2^)** | **p-value** | **Slope of the regression model** |
| --- | --- | --- | --- |
| Visual | 0.063 | 0.023 | -0.040 |
| Somatomotor | 0.115 | 0.002 | -0.097 |
| Dorsal Attention | 0.103 | 0.003 | -0.071 |
| Ventral Attention | 0.069 | 0.017 | -0.080 |
| Limbic | 0.195 | 0.001 | -0.103 |
| Frontoparietal | 0.040 | 0.072 | -0.059 |
| Default Mode | 0.023 | 0.174 | -0.052 |

**Figure S1:** Regression analyses for the entire patient cohort between the mean functional connectivity (z-score) of the metabolically active tumor region and its proximity (mm) to the dorsal attention network. Abbreviations: R^2^, coefficient of determination; N, number of patients

**References**

**1.** Herzog H, Langen KJ, Weirich C, et al. High resolution BrainPET combined with simultaneous MRI. *Nuklearmedizin.* 2011; 50(2):74-82.
